# Immunomodulatory role of histamine H4 receptor in breast cancer

**DOI:** 10.1038/s41416-018-0173-z

**Published:** 2018-07-10

**Authors:** Helena A. Sterle, Melisa B. Nicoud, Noelia A. Massari, Mónica A. Táquez Delgado, María V. Herrero Ducloux, Graciela A. Cremaschi, Vanina A. Medina

**Affiliations:** 1 Neuroimmunomodulation and Molecular Oncology Division, Institute for Biomedical Research (BIOMED), School of Medical Sciences, Pontifical Catholic University of Argentina (UCA), and the National Scientific and Technical Research Council (CONICET), Buenos Aires, Argentina; 2 Laboratory of Tumor Biology and Inflammation, Institute for Biomedical Research (BIOMED), School of Medical Sciences, Pontifical Catholic University of Argentina (UCA), and the National Scientific and Technical Research Council (CONICET), Buenos Aires, Argentina; 3 Laboratory of Radioisotopes, School of Pharmacy and Biochemistry, University of Buenos Aires, Buenos Aires, Argentina; 4 Immunology Department, School of Natural Sciences, National University of Patagonia San Juan Bosco, Chubut, Argentina; 5 Pathology Department, School of Natural Sciences, National University of Patagonia San Juan Bosco, Chubut, Argentina

**Keywords:** Immunoediting, Breast cancer

## Abstract

**Background:**

Although the role of histamine H4 receptor (H4R) in immune cells is being extensively investigated, its immunomodulatory function in cancer is completely unknown. This study aimed to investigate the role of H4R in antitumour immunity in a model of triple-negative breast cancer.

**Methods:**

We evaluated growth parameters, histological characteristics and the composition of tumour, splenic and tumour draining lymph node (TDLN) immune subsets, in a syngeneic model, developed orthotopically with 4T1 cells in H4R knockout (H4R-KO) and wild-type mice.

**Results:**

Mice lacking H4R show reduced tumour size and weight, decreased number of lung metastases and percentage of CD4^+^ tumour-infiltrating T cells, while exhibiting increased infiltration of NK cells and CD19^+^ lymphocytes. Likewise, TDLN of H4R-KO mice show decreased CD4^+^ T cells and T regulatory cells (CD4^+^CD25^+^FoxP3^+^), and increased percentages of NK cells. Finally, H4R-deficient mice show decreased Tregs in spleens and non-draining lymph nodes, and a negative correlation between tumour weight and the percentages of CD4^+^, CD19^+^ and NK splenic cells, suggesting that H4R also regulates antitumour immunity at a systemic level.

**Conclusions:**

This is the first report that demonstrates the participation of H4R in antitumour immunity, suggesting that H4R could be a target for cancer treatment.

## Introduction

Cancer is a major public health issue, representing one of the main causes of death in the world. Breast cancer is the neoplasm with the highest incidence among women both in developed countries and in developing ones.^1^ During carcinogenesis, the transformed cells interact with their microenvironment comprising stromal and immune cells.^2^

A large body of data revealed the importance of the leukocyte infiltrate in controlling the clinical progression of various types of epithelial cancers.^3^ Cells of immune system are key constituents of the tumour microenvironment and may promote or inhibit the development and progression of cancer.^4^ One of the common features of most tumour processes is the inflammation that can contribute to proliferation and survival of malignant cells, promote angiogenesis and metastasis, altering the responses to hormones and chemotherapeutic agents.^2^ On the other hand, the immune system through tumour immune surveillance, is also responsible for the removal of nascent tumour cells, preventing tumour progression and formation of micrometastases.^2,5^ There are also immunosuppressive mediators in the tumour microenvironment that regulate the activity and efficiency of cells with antitumour capacity, thus controlling the elimination of tumour cells. In this regard, immunotherapy has emerged as a useful therapeutic approach for the treatment of many cancers as it stimulates the immune system to detect and eradicate tumour cells.

Breast cancer was traditionally considered poorly immunogenic and therefore, immunotherapeutic approaches were not considered appropriate for its treatment. However, recent studies have shown that the presence of tumour-infiltrating lymphocytes (TILs) is a prognostic marker for higher responses to neoadjuvant chemotherapy and better survival, particularly in triple-negative breast cancer (TNBC).^6^ Breast carcinomas are frequently infiltrated by inflammatory cells, mainly macrophages and T lymphocytes, whose significance has not yet been fully elucidated.^7^ Several studies agree, however, that the number of infiltrating CD8^+^ cells correlates with better patient survival and response to chemotherapeutic treatments.^8^ B cells have also been associated with a favourable prognosis in breast cancer, although its mechanism of action in cancer is still under debate.^9^ In contrast, CD4^+^ T lymphocytes, which include regulatory T cells (Treg) and tumour associated macrophages, are associated with poorer clinical outcomes. Thus, the analysis of the tumour infiltrating leukocytes and the study of different agents with the ability to modulate the immune response are currently under consideration, mainly for the treatment of triple negative breast cancer, that is not only unresponsive to targeted therapies but also less responsive to conventional therapeutics.^6,10^

There is an increasing evidence that supports the immunomodulatory roles of histamine under different cytokine environments^11^ and it was also described as an important modulator of haematopoiesis.^12^ Histamine is a biogenic amine and is one of the most general biological mediators of mammals, presenting numerous physiological and pathophysiological functions, which it exerts through the activation of four different receptor subtypes H1, H2, H3 and H4 receptors (H1R, H2R, H3R and H4R), which belong to the family of seven transmembrane domain G-protein-coupled receptors.^12,13^ Histamine treatment stimulates the maturation of dendritic cells from monocytes and promotes the intratumoural accumulation of these cells, reducing the growth of murine lymphoma developed with EL-4 cells.^14^ However, in a histidine decarboxylase knockout model, endogenous histamine contributed to the growth of a breast tumour by suppressing the antitumour immunity.^15^ Therefore, different histamine metabolism, distinct tumour microenvironment and the histamine receptor that is involved, may determine the outcome.

H4R is the last discovered member of the histamine receptor family and is mainly expressed in cells of the immune system, such as mast cells, eosinophils, monocytes, dendritic cells, T lymphocytes and Natural Killer (NK) cells. In peripheral tissues such as the spleen, thymus and bone marrow its levels of expression are altered in response to inflammatory stimuli.^16^ Several lines of evidence indicate that the H4R plays a key role in both pulmonary and intestinal inflammatory diseases (asthma, colitis), pruritus, allergies and other immunological disorders, which has led to the therapeutic development of novel H4R ligands. This is supported by results obtained in different experimental models of inflammation including hepatic ischaemia-reperfusion, colitis, atopic dermatitis, in which H4R antagonists (JNJ7777120, JNJ10191584, thioperamide) produce anti-inflammatory effects with reduced neutrophil recruitment and release of cytokines, improving the outcome of the pathology.^17,18^ In some of these models H4R antagonists produce the same phenotype observed in H4R knockout mice. Recent clinical trials show that selective H4R antagonists reduce pruritus in patients with atopic dermatitis and improve allergic rhinitis, validating preclinical results.^17,18^

Although the role of H4R in immune cells during inflammatory disorders is being extensively investigated, its immunomodulatory function in cancer is completely unknown. This study aimed to investigate the role of H4R in antitumour immunity in a model of TNBC. We evaluated growth parameters, histological characteristics of tumours and the composition of tumour, splenic and tumour draining lymph node (TDLN) immune subsets in a syngeneic model of TNBC developed orthotopically with 4T1 cells in H4R knockout (H4R-KO) and wild-type (WT) mice.

Results show an interplay between H4R expressing immune cells in tumour microenvironment and cancer cells, which has implications in breast cancer progression.

## Materials and methods

### Cell culture

The tumour cell line 4T1 (ATCC CRL-2539) was cultured and was maintained in Dulbecco’s Modified Eagle Medium (DMEM) supplemented with 10% (v/v) FBS, 0.3 g/l glutamine, 100 µg/ml streptomycin, and 100 U/ml penicillin (all from Gibco BRL, Grand Island, NY, USA). Cells were maintained at 37 °C in a humidified atmosphere containing 5% CO_2_.

### H4R expression

H4R expression in 4T1 cells was determined by RT-PCR and immunostaining. The retrotranscription reaction and RT-PCR were performed as previously described.^19^ Negative controls were performed with water instead of cDNA and cDNA of H4R-KO mice spleen. H4R primers and PCR conditions were as followed: H4R-F: GGC CAA GTG GAT CTC CTG TA, H4R-R: GCT CTG AAA GCA ACC TAA CTG TG, 500 bp; 3 min 94 °C, 35 cycles of 30 s 94 °C, 30 s 62 °C, 30 s 72 °C. β-actin was used as load control. PCR products were subjected to gel electrophoresis and visualised using BioDoc-It Imaging System (USA).

Immunostaining was done as reported.^19^ Briefly, cells grown on coverslips were fixed and then incubated overnight in a humidified chamber at 4 °C with primary goat anti-H4R (1:100, catalogue number SC-33967, Santa Cruz, USA). After washing, cells were incubated with FITC-conjugated anti-goat (1:400), and nuclei were counterstained with Dapi (Sigma Chemical Co., USA) at room temperature. Coverslips were mounted with FluorSave^TM^ Reagent (Calbiochem, USA) and fluorescence was evaluated by using a Axiovert 200 microscope (Carl Zeiss, Germany).

### Animal models

Female histamine H4 receptor knockout (H4R-KO) were generated as previously described^20^ and were provided by Janssen Research & Development, LLC La Jolla, CA, USA and back crossed to BALB/c background. These animals and the corresponding female wild-type (WT) mice (BALB/c) were bred and kept in ventilated cages at our animal health care facility at 22–24 °C and 50–60% humidity on a 12 h light/dark cycle with food and water available ad libitum. Animals with an age of 6–8 weeks and an average weight of 20–25 g were used. All animal protocols were supervised and managed by qualified trained personnel and were in accordance with recommendations from the National Institute of Health Guide for the Care and Use of Laboratory Animals (NIH Publications No. 8023) and the Guidelines for the welfare and use of animals in cancer research. All procedures involving animals were reviewed and approved by the Institutional Committee for the Care and Use of Laboratory Animals, BIOMED.

### Breast cancer model

To generate solid tumours, 6 to 8-week-old mice were inoculated orthotopically in the abdominal mammary gland with 1 × 10^5^ syngeneic 4T1 cells in serum-free PBS, as described.^21,22^ Tumour length and width were measured every 2 days using callipers, and tumour volume was calculated as *V* = *π*/ 6 × length × width.^2,23^ After 21 or 28 days, mice were sacrificed, and tissues were removed and weighted. When indicated, WT mice were daily treated with subcutaneous injections of JNJ28610244 (JNJ28, 5 mg/kg b.w.), (Janssen Research & Development, San Diego, USA) for the last 14 days.

### Preparation of single-cell suspensions from lymph nodes, spleens and tumours

After removal, lymphoid organs were disrupted through a 1-mm metal mesh. Tumours were minced and digested with 2 mg/ml collagenase type I (Gibco) in serum-free DMEM for 30 min at 37 °C. After centrifugation, the red blood cells from the resulting cell suspensions were lysed using a buffer containing 150 mM NH_4_Cl, 10 mM K_2_CO_3_ and 0.1 mM EDTA and then filtered through a 40-µm cell strainer (BD Biosciences, San José, CA, USA) and resuspended in PBS.^22,23^

### Flow cytometry for immunophenotyping

Single-cell suspensions obtained from tumours, tumour draining (TDLN) and non-draining lymph nodes and spleens were stained with antibodies against various cell surface markers using standard staining methods, as previously described.^22,23^ The following panel of commercially available and fluorochrome conjugated anti-mouse monoclonal antibodies were used in the study: FITC-anti-mouse CD3 (BDB-561798), FITC-anti-mouse CD4 (BDB-557307), PE-anti-mouse CD4 (BDB-557307), PE-anti-mouse CD8 (BDB-553032), PE-anti-mouse CD19 (BDB-557399), FITC-anti-mouse Gr1 (BDB-553126), PE-anti-mouse CD11b (BDB-557397), FITC-anti-mouse CD49 (BDB-561066), FITC-anti-mouse CD44 (BDB-561859) and APC-anti-mouse CD25 (BDB-558643), all purchased from BD Biosciences (BDB; San José, CA, USA). Samples were run on a BD Accury C6 flow cytometer (BDB) and data were analysed using the BD Accury C6 software (BDB).

### Intracellular staining

Single cell suspensions, prepared as described above, were used for intracellular staining. After surface staining, cells were fixed with Mouse Fixation Buffer (BD, 51-9006124) and permeabilised with Mouse Foxp3 Permeabilization Buffer (BDB, 51-9006125), following manufacturers’ instructions. Cells were then incubated with the FITC-anti-mouse-FoxP3 antibody (BDB-560408) for 40 min. After washing with PBS, cells were analysed by flow cytometry as described above.

### Cytokine determination

Tumours, TDLN and spleens were obtained from mice 28 days post-tumour inoculation (p.i.). Lymphoid organs were disrupted through a 1-mm metal mesh and seeded at a final concentration of 1 × 10^7^ cells/ml in complete RPMI medium. Tumours were cut in small pieces and equal quantities of tissue were incubated in complete RPMI medium. After 24 h, the conditioned medium was obtained. Mice interferon (IFN)-γ (BDB-558473), tumour necrosis factor (TNF)-α (BDB-558480) and IL-10 (BDB-558300) CBA Flex Sets (BD Biosciences) were used to analyse specific cytokines in a BD Accury C6 flow cytometer, following the manufacturers’ instructions. Results were analysed with FCAP Array Software v3.0 (BDB).

### Histochemistry and immunostaining

Tumours and tissues were excised, fixed in 4% (v/v) formaldehyde in PBS (formalin), paraffin embedded and sliced into 3–4-μm-thick sections to evaluate the histological characteristics on haematoxylin–eosin (H&E) stained specimens (Biopur diagnostic, Buenos Aires, Argentina) as previously described.^19^

Cell proliferation was assessed by histochemical determination of PCNA (1:100, clone PC10, Dako Cytomation, Denmark) expression and by mitotic index (MI), as the number of cells with visible chromosomes in ×400 magnification fields. Intratumoural vascularity was evaluated by counting vessels at ×200 magnification in 10 random fields. The fragmented DNA was detected using Apoptag^TM^ plus peroxidase in situ apoptosis Detection Kit (Millipore, MA, USA) according to the manufacturer’s instructions. These procedures were described in detail previously.^19^

Metastatic spread analysis was performed over cuts of excised organs after staining with H&E. Visualisation was performed with an optical microscope (Axiolab Carl Zeiss, Germany) and photographs were taken at ×200 or ×630 magnification with Canon Power Shot G5 camera (Japan). The immunostaining assessment was blinded performed by consensus agreement of two observers.

### Statistical analysis

Representative results are presented as means ± standard error of the mean (SEM). Statistical evaluations were made by unpaired *t*-test or analysis of variance (ANOVA) that was followed by Newman–Keuls Multiple Comparison Test. Pearson’s *r* correlation coefficient and two-tailed significances were determined when appropriate. All statistical analyses were performed with GraphPad Prism version 6.00^™^ (CA, USA).

## Results

### H4R-KO mice exhibit reduced tumour growth and metastasis

**Fig. 1:**
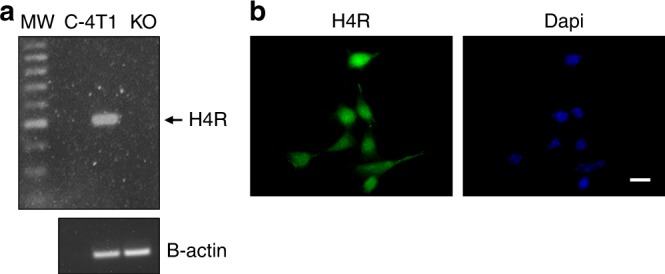
H4R expression was evaluated in 4T1 cells by RT-PCR (**a**) and immunostaining (**b**). **a** Lanes: MW, DNA ladder molecular size marker; C- water replace cDNA, 4T1: cDNA of 4T1 cells, KO: spleen cells of H4R-KO mice were used as negative control. β-actin (521 bp) was used as load control. **b** Immunofluorescence (green) of H4R in 4T1 cells. Nuclei were counter-stained with Dapi (blue). Scale bar = 20 µm

**Fig. 2:**
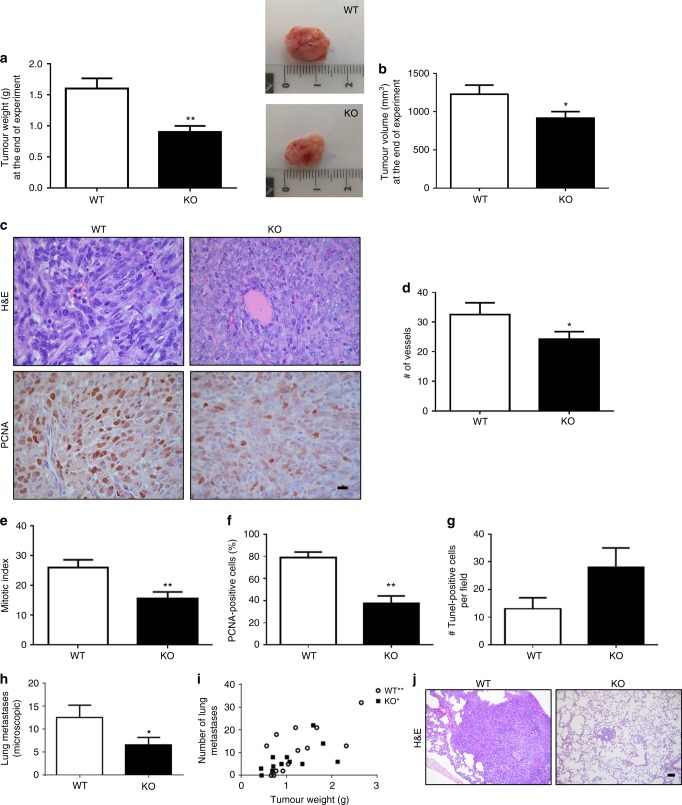
Tumour growth parameters of 4T1 tumour-bearing WT and H4R-KO mice. Comparison of (**a**) tumour weight and (**b**) tumour volume at the end of the experimental period (28 days). Inset: representative pictures of tumours. **c**, **j** Representative H&E images of paraffin-embedded (**c**) tumours and (**j**) lungs specimens. **c** Representative pictures of PCNA-positive immunostaining of tumours (×630 and ×200 original magnification, Scale bar = 20 µm). **d** Number of vessels: number of intratumoural vessels at ×200 magnification in 10 random fields (hot spots). **e** Mitotic index, number of cells with visible chromosomes at ×400 magnification in 5 random fields. **f** Percentage of PCNA-positive cells per field and (**g**) number of TUNEL-positive cells per field at ×400 magnification in 10 random fields. **h** Number of microscopic metastatic foci covering lungs. Error bars represent the means ± SEM of three independent experiments (*T*-Test, *P < 0.05, **P < 0.01 vs. WT). **i** Pearson’s correlation between the number of lung metastases and the tumour weight (correlation coefficient, *r*: 0.6851, **P = 0.0098 in WT mice. *r*: 0.5976, *P = 0.0402 in KO mice)

In agreement with previous studies in human breast cancer cell lines,^24,25^ the expression of H4R in 4T1 cells was demonstrated by RT-PCR and immunostaining (Fig. 1a, b). To investigate the effect of the H4R-expressing cells in tumour microenvironment on breast cancer development and progression, H4R-KO and WT mice were injected orthotopically with 4T1 cells. Both sets of mice developed tumours, although H4R-KO mice displayed significantly reduced endpoint tumour size and weight compared to WT mice (Fig. 2a, b). Histopathological analysis revealed that H4R-KO mice exhibited areas of tubular differentiation and reduced nuclear pleomorphism together with decreased mitotic index and PCNA proliferation marker expression, whereas WT mice exhibited higher undifferentiation (Fig. 2c, e, f). Consistent with these results, tumours developed in H4R-KO mice showed increased apoptosis and decreased vascularisation along with a reduced number and size of lung micrometastasis compared to tumours of WT animals (Fig. 2d, g, h, j). In addition, a positive correlation between tumour weight and the number of lung metastases was observed in both WT and H4R-KO mice (Fig. 2i).

**Fig. 3:**
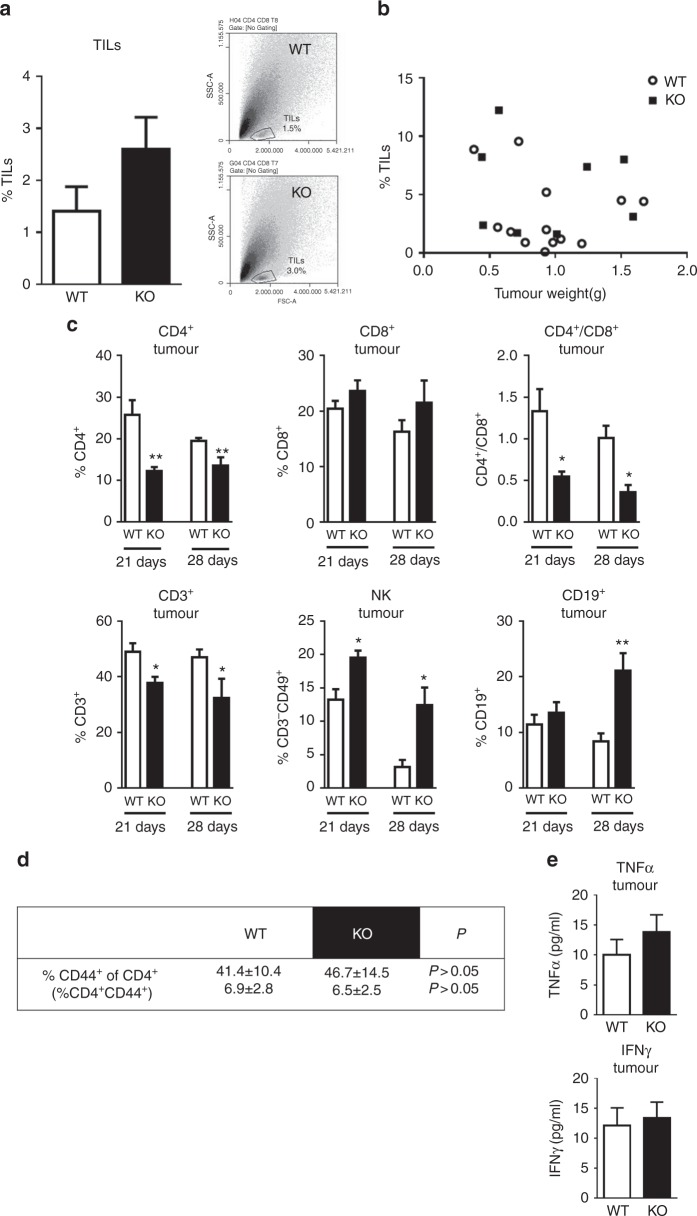
Distribution of immune cell subsets in tumours of 4T1 cells developed in WT and H4R-KO mice. **a** %TILs: Tumour-infiltrating lymphocytes percentage. Inset: dot plot: SSC-A vs. FSC-A. **b** Pearson correlation of TILs vs. tumour weight (*P*>0.05). **c**, **d** Tumour cell suspensions were obtained 21 or 28 days p.i. of 4T1 cells and were labelled with specific antibodies: CD4-FITC: T helper lymphocytes marker, CD8-PE: T cytotoxic lymphocytes marker, CD19-PE: B lymphocytes marker, CD49-PE and CD3-FITC: NK markers, CD3-FITC: T lymphocytes marker, CD44-FITC: activated T lymphocyte marker. Ratio CD4^+^/CD8^+^. Error bars represent the means ± SEM of three independent experiments (*T*-test, *P < 0.05, **P < 0.01, ***P < 0.001 vs. WT). **e** Cytokine concentration in tumour-conditioned medium. Error bars represent the means ± SEM of two independent experiments

Considering the pivotal role of immunity in tumour microenvironment and that H4R is primarily expressed on immune cells, the inflammatory infiltrate was next investigated. Tumour-infiltrating lymphocytes (TILs) were evaluated by FACS. Although not significant, a higher percentage of TILs was observed in tumours of H4R-KO mice, whereas no correlation was observed between TILs and tumour weight (Fig. 3a, b). The analysis of the distribution of the tumour-infiltrating immune cell subsets was performed 21 and 28 days post-tumour inoculation and it showed decreased CD3^+^ tumour-infiltrating lymphocytes in H4R-KO mice. However, no changes in the CD8^+^ T cell subset were detected, but a decreased percentage of CD4^+^ T cells in this group lead to a reduced CD4^+^/CD8^+^ ratio. This was accompanied by increased percentages of NK cells from day 21 in tumours of H4R-KO compared to WT mice. Also increased CD19^+^ lymphocytes were detected in these tumours that became evident only at day 28 (Fig. 3a, c).

CD44 is a marker of T cell activation and its high expression is one of the most reliable markers for both CD4^+^ and CD8^+^ memory T cells in mice.^26^ No significant differences were observed in the percentage of CD4^+^CD44^+^ (Fig. 3d) or CD8^+^CD44^+^ T lymphocytes (data not shown) in both groups of animals. Interestingly, further correlation analysis demonstrated a positive association of CD8^+^ lymphocytes with tumour weight, exclusively in H4R-KO mice (Supplementary Table 1). The analysis of cytokine production in the conditioned medium of tumours showed no differences in the levels of IFNγ, but a non-significant increase in TNFα levels in tumours of H4R-KO mice (Fig. 3e).

### H4R is involved in the regional and systemic antitumour immunity

**Fig. 4:**
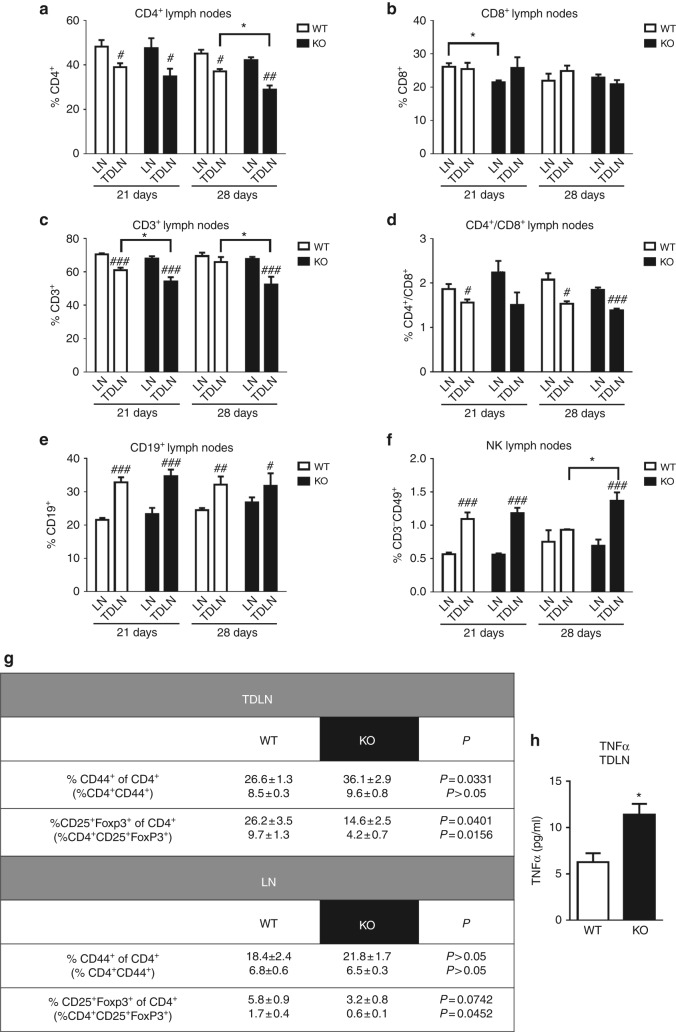
Distribution of immune cell subsets in tumour draining (TDLN) and non-draining lymph nodes (LN) of 4T1-tumour bearing Balb/c mice. Cell suspensions from lymph nodes obtained from 21- or 28-day-tumour bearing mice were labelled with specific antibodies: **a** CD4-FITC: T helper lymphocytes marker, **b** CD8-PE: T cytotoxic lymphocytes marker, **c** CD3-FITC: T lymphocytes marker, **d** ratio CD4^+^/CD8^+^, **e** CD19-PE: B lymphocytes marker, **f** CD49-PE and CD3-FITC: NK markers, **g** CD44-FITC: activated T lymphocyte marker; CD4-FITC, CD25-APC, FoxP3-PE: Treg markers. Error bars represent the means ± SEM of three independent experiments (ANOVA and Newman–Keuls Multiple Comparison Test. *P < 0.05 vs. WT; *T*-test, ^#^*P* < 0.05, ^##^*P* < 0.01, ^###^*P* < 0.001 vs. LN). **h** TNFα concentration in TDLN-conditioned medium. Error bars represent the means ± SEM of two independent experiments (*T*-test, *P < 0.05)

We next evaluated the role of the H4R in the regional and systemic immune response by analysing, in WT and H4R-KO mice, the distribution of immune cells in tumour-draining lymph nodes (TDLN) and non-draining lymph nodes (LN), respectively. No significant differences were observed in the percentage of T (CD3^+^), B (CD19^+^) or NK (CD3^-^CD49^+^) cells in LN in both groups (Fig. 4a–f). However, TDLN of WT and H4R-KO mice showed significant differences in the percentage of these immune cell subsets. At day 21 p.i., TDLN of H4R-KO mice showed a decreased percentage of CD3^+^ lymphocytes. A reduction of CD4^+^ cells and increased percentage of NK cells compared to WT mice became evident at day 28 p.i. of 4T1 cells, while no differences in the proportion of CD8^+^ T or CD19^+^ B lymphocytes were observed (Fig. 4a–f). However, the percentage of CD4^+^ activated T lymphocytes was increased in TDLN of H4R-KO mice, as it could be determined through CD44 staining in these cells (Fig. 4g). H4R-KO mice also showed a reduced percentage of CD4^+^CD25^+^FoxP3^+^ Treg cells in both TDLN and LN when compared with WT mice (Fig. 4g).

Interestingly, a reduced percentage of CD4^+^ T lymphocytes and increased CD19^+^ B lymphocytes and NK cells were observed in TDLN compared to non-draining nodes in both WT and H4R-KO mice (Fig. 4a–f).

As it was observed in tumours, a positive correlation between the percentage of CD8^+^ T lymphocytes in TDLN and tumour weight was detected. On the other hand, a negative association between the percentage of NK and CD19^+^ lymphocytes in TDLN and tumour weight was observed exclusively in H4R-KO mice (Supplementary Table 2). Additionally, increased levels of TNFα were measured in conditioned medium of TDLN from H4R-KO mice, compared to WT, while IFNγ levels were under detection limits (Fig. 4h).

Spleen regulates innate and adaptive immune responses, being an essential organ in the systemic antitumour immunity.^27^ Therefore, the spleens from tumour-bearing animals were further examined. In line with previous literature,^28^ a significant increase in spleen size and weight was observed in 4T1 tumour bearing animals (Table 1) compared to spleens of mice without tumours (WT: 0.09 ± 0.01 g, KO: 0.10 ± 0.01 g). Splenomegaly develops in response and is directly correlated to tumour growth.^28^ In agreement with this statement, spleens of tumour-bearing H4R-KO mice demonstrated a reduced weight in comparison with the ones of WT mice (Table 1). Similar histologic characteristics, including red and white pulp areas, were observed in spleens derived from tumour-bearing H4R-KO and WT mice. Red pulp areas revealed prominent megakaryocytes and granulocytes (data not shown).

**Table 1:** Distribution of splenic immune cell subsets and spleen weight of 4T1 tumour-bearing WT and H4R-KO mice

	WT	KO	
% CD3^+^	13.5±1.3	14.3±1.0	*P*>0.05
% CD4^+^	8.8±0.7	9.1±0.4	*P*>0.05
% CD8^+^	6.4±0.6	6.5±0.3	*P*>0.05
% CD3^-^CD49^+^	2.8±0.4	3.0±0.1	*P*>0.05
% CD19^+^	24.6±2.2	26.5±1.6	*P*>0.05
% CD4^+^/CD8^+^	1.5±0.3	1.6±0.3	*P*>0.05
% CD44^+^ of CD4^+^ (% CD4^+^CD44^+^)	30.0 ± 3.4 (3.9 ± 0.7)	31.5±3.9 (3.8±1.0)	*P* > 0.05 (*P* > 0.05)
% CD25^+^FoxP3^+^ of CD4^+^ (% CD4^+^CD25^+^FoxP3^**+**^)	2.4 ± 0.4 (0.34 ± 0.09)	1.1 ± 0.1 (0.09±0.02)	*P*=0.0793 (*P*=0.0792)
Spleen weight (g)	0.61±0.06	0.43±0.03	*P*=0.0170

Splenic cell suspensions were obtained from 28-day tumour-bearing mice and were labelled with specific antibodies: CD3-FITC: T lymphocytes marker, CD4-FITC: T helper lymphocytes marker, CD8-PE: cytotoxic T lymphocytes marker, CD49-PE and CD3-FITC: NK markers, CD19-PE: B lymphocytes marker, CD44-FITC: activated T lymphocyte marker, CD4-FITC, CD25-APC, FoxP3-PE: Treg markers. Error bars represent the means ± SEM of three independent experiments (*T*-Test)

Furthermore, the distribution of splenic immune cell subsets was determined in the spleens of 4T1 tumour-bearing WT and H4R-KO mice. In spite of the negative correlations between the tumour weight and the percentages of CD3^+^ T, CD4^+^ T and CD19^+^ B lymphocytes and NK cells in spleen that were found only in H4R-KO mice at day 28 p.i. (Supplementary Table 3), no differences were found in the percentages of CD4^+^ T or CD8^+^ T, CD19^+^ B, NK cells in spleens of tumour bearing H4R-KO mice compared to WT mice (Table 1). However, splenic Tregs were decreased in H4R-KO mice (Table 1). Both TNFα and IFNγ levels in conditioned medium of spleens were found undetectable (data not shown).

### H4R stimulation enhances immunosuppression in 4T1 breast tumours

**Fig. 5:**
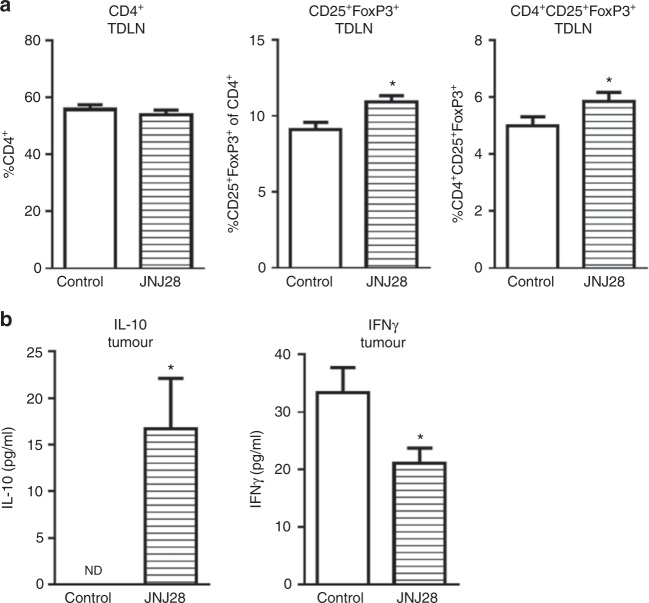
Treatment of tumour-bearing WT mice with the H4R agonist JNJ28610244, JNJ28 (5 mg/kg b.w.). **a** Cell suspensions from TDLN obtained from 28-day tumour-bearing mice were labelled with specific antibodies: CD4-FITC, CD25-APC, FoxP3-PE. **b** IL-10 and IFNγ cytokine concentration in tumour-conditioned medium. Error bars represent the means ± SEM of two independent experiments (*T*-Test, *P < 0.05)

To further investigate the immunosuppressive role of H4R, WT mice were treated with the H4R agonist JNJ28. Even though the treatment had no effect on the distribution of total CD4^+^ lymphocytes in TDLN, the percentage of CD4^+^CD25^+^FoxP3^+^ Treg cells was increased in treated mice (Fig. 5a). Moreover, increased levels of interleukin (IL)-10 were detected in tumours of treated mice (Fig. 5b), that were accompanied by decreased levels of IFNγ (Fig. 5b).

## Discussion

During carcinogenesis, the interaction between cancer cells and immune system remodels the tumour microenvironment to impair the host immune response, which is recognised as one of the hallmarks in cancer.^2^ The identification of the molecular pathways involved in the dysregulation of tumour immunity is of outmost importance for the improvement of cancer therapy.

The H4R is the latest characterised histamine receptor and it is mainly expressed on cells of the haematopoietic lineage.^29,30^ In this regard, H4R modulates important immune functions in both innate and adaptive immune responses.^31–33^ Reports on the H4R in mast cells, NK, eosinophils, T cells, monocytes and dendritic cells demonstrated numerous immune modulating physiological functions of this receptor, including chemotaxis and pro-inflammatory cytokine and chemokine release.^20,30,34–38^ Accordingly, the receptor is implicated in the pathology of various immune system-associated diseases such as autoimmune disorders, bronchial asthma and pruritus,^39^ which highlights the therapeutic potential of H4R ligands in inflammatory and autoimmune disorders,^40,41^ and some of them have reached clinical scenarios for the treatment of atopic dermatitis and pruritus.^18^

The expression of H4R and histamine-induced modulation of proliferation in different types of tumours has been previously reported.^12,42,43^ Functional expression of H4R has been demonstrated in human breast cancer tissues and cell lines, exhibiting a key role in histamine-mediated biological processes associated with cancer progression. In vivo treatments with histamine or H4R agonists diminished the growth rate of human TNBC developed in immune-deficient nude mice with MDA-MB-231 cells.^43,44^

In agreement with these results, the H4R agonist 4-methylhistamine significantly decreased the tumour volume and increased the survival of mice bearing xenograft non-small cell lung cancer tumours.^45^ In addition, H4R agonists significantly increased the median survival and decreased the tumour volume in vivo in two human melanoma experimental models.^19,42^ However, all these studies were performed in immunodeficient hosts, where the role of the immune system in the response to anti-cancer treatments and tumour progression could not be evaluated and therefore, they may not mimic the real clinical situation.

Although it is well established that the immune tumour microenvironment is a major participant in cancer cell proliferation, invasion and metastasis; the role of H4R in the antitumour immunity is completely unknown. In the present study we aimed to investigate the immunomodulatory role of H4R in breast cancer, evaluating growth parameters and the composition of tumour, splenic and TDLN immune subsets in a syngeneic model of TNBC developed orthotopically with 4T1 cells in H4R-KO and WT mice. The 4T1 tumour model in immunocompetent mice resembles human TNBC as it leads to metastasis formation in various organs, including the lung, brain and bone and is widely used for the preclinical determination of antitumour therapies to evaluate the role of the immune system in the tumour development and progression.^22,46^

Results show that H4R selectively affects the distribution of different immune cell populations in the tumour microenvironment, modulating the local and systemic immune responses. Hosts with H4R deficiency exhibited reduced tumour growth that was correlated with a reduced number of lung metastasis. This fact could determine the disease outcome, as metastasis is the primary cause of death in breast cancer patients. Metastasis formation is regulated not only by the tumour cells but also by unique molecular signals from the tumour microenvironment^47^ and the immune response induced by the primary tumour, which plays important roles in all steps of the metastatic cascade.^48^

The reduced tumour growth was associated with a decreased angiogenesis and different immune cell infiltration. It is well documented that tumour growth is strongly related to neovascularisation. In this work, a reduced number of intratumoural vessels was observed in H4R-KO mice, which could contribute to the decreased size and proliferation of these tumours. In line with this study, laser choroidal neovascularisation volume was reduced in H4R-KO mice.^47^ On the other side, other reports demonstrated that H4R may negatively regulate angiogenesis, since H4R agonists attenuate angiogenesis in Leydig tumours^48^ and melanoma.^44^ The study of the exact role of H4R in tumour angiogenesis is still unexplored and deserves further investigation.

On the other hand, the outcome of the antitumour immunity is determined by the type of the developed immune response and TILs, including NK and cytotoxic CD8^+^ T cells, are known to protect against tumour development and progression.^49^ In this regard, TILs in human biopsies are emerging as a positive prognostic factor in different solid tumours, including breast cancer.^50^ In addition, TDLN are used as prognostic markers in breast cancer. They have a dual role in tumour development, as they can induce antitumour immune responses but they can also act as routes for malignant cells towards distant organ metastasis in response to different factors released by tumours.^51^

Our results show a decreased ratio of CD4^+^/CD8^+^ lymphocytes in tumours and TDLN of H4R-KO mice that could contribute for the slower growth rate of these tumours. In fact, CD8^+^ T-cell infiltrates are generally associated with better prognosis while CD4^+^ T cells, which include T-regulatory cells, have been associated with worse outcomes.^8^ Immunohistochemical analysis of tissue microarrays derived from 179 treatment-naive breast tumours revealed that high levels of CD4^+^ T cells correlated with reduced overall survival (OS), while high levels of CD8^+^ T cells combined with low levels of CD4^+^ T cells correlated with increased OS.^52^

It is known that T lymphocytes express H4R and that its expression is higher in resting than in activated T cells.^53^ However, very little is known about the role of this receptor in T cells. Gantner et al.^11^ described that H4R, together with H2R, are involved in the control of IL-16 release from human CD8^+^ T cells.^11^ IL-16 is believed to play an important role in the recruitment of CD4^+^ T cells mainly in inflammatory diseases, but it is also involved in breast cancer and other tumours.^54–56^ In a murine model of allergic inflammation, H4R-KO mice showed reduced lung infiltrating T cells accompanied with decreased Th2 responses.^57^ Moreover, in a chronic model of atopic dermatitis, H4R-KO mice showed amelioration of the skin lesions and displayed a reduced number of splenocytes and lymph node cells with a decreased number of CD4^+^ T cells.^58^ In this model, the H4R also modulated the cytokine secretion of CD4^+^ T cells and splenocytes and altered the cellular profile in the lymph nodes. Additionally, the treatment with the H4R agonist 4-methylhistamine in a murine model of chronic stress has been shown to increase the number of CD4^+^ cells and the production of Th1 cytokines in whole blood.^59^

A more exhaustive analysis on CD4^+^ cells showed no differences in the percentage of activated CD4^+^ infiltrating lymphocytes and no Tregs could be detected in tumours. On the other hand, TDLN of H4R-KO exhibited reduced Tregs compared to WT mice. Although TDLN of H4R-KO mice showed a higher percentage of activated cells within CD4^+^ subset, total activated CD4^+^ cells were similar in both groups. It is important to highlight the increased presence of Tregs in TDLNs compared to non-draining LN in both groups. This has been well established in both animal models and cancer patients, where accumulation of Tregs in TDLNs and not in tumour is correlated with disease progression.^51^ In addition, treatment with a specific H4R agonist, JNJ2828610244, significantly increased the percentage of Tregs in TDLN of WT mice. IL-10 is a well-known immunosuppressive cytokine, which is secreted by tumour cells and Tregs among other immune cells and has an important role in tumour immunosurveillance.^60^ On the other hand, IFNγ is a pleiotropic molecule with antitumour effects, which is produced primarily by cytotoxic CD8^+^ T lymphocytes and NK cells.^61^ H4R stimulation enhanced IL-10 while decreased IFNγ levels in tumour conditioned medium of WT mice, suggesting an immunosuppressive role of H4R in the context of 4T1 breast cancer model.

In line with these results, it has been previously demonstrated that H4R regulates Tregs activity. In a murine model of allergic asthma, the treatment with the H4R agonist 4-methylhistamine resulted in accumulation of FoxP3^+^ T cells with potent suppressive activity for proliferation.^62^ Likewise, H4R was described to play a critical role in determining the frequency of Tregs in secondary lymphoid tissues in a model of allergic encephalomyelitis, as it regulates Treg chemotaxis and suppressor activity. Moreover, the lack of H4R leads to an impairment of an anti-inflammatory response due to fewer Tregs in the central nervous system during the acute phase of the disease.^63^ Tregs have been described to limit NK cell numbers by direct granzyme B and perforin-dependent killing in TDLN.^64^ NK cells perform several important functions, including the regulation of the adaptive immune response by secreting cytokines like IFNγ and producing the lysis and destruction of tumour cells.^35^ NK cells constitutively express a lytic machinery capable of killing target cells independently of antigen presentation.^65^ The expression of the H4R in NK cells was determined at the protein level^35^ and the stimulation of these cells with the H4R agonist ST1006 showed increased production of CCL3^66^ and induced NK cell chemotaxis in vitro, which was inhibited with the H4R antagonist JNJ7777120.^67^ However, our results show an increased percentage of NK cells in tumours and TDLN of H4R-KO mice when compared with WT mice, indicating an increased chemotaxis to the tumour site of these cells in mice lacking the H4R. This effect should be an early event in tumour development, considering that the increase in NK tumour infiltration is evident at 28 but also 21 days post 4T1 cells’ injection. These animals also showed increased levels of TNFα in TDLN and, although no significant, in tumours, which could be related to NK cell activity, even though no differences were observed in the production of IFNγ.

In agreement with present results, NK cells were shown to play important roles in tumour immune surveillance, in the prevention of tumour progression and against metastatic dissemination in numerous murine models.^68,69^ Most human tumours display low levels of NK cells and their increased infiltration is associated with an improved prognosis and reduction of tumour recurrence.^68^ Surprisingly, the increase in tumour-infiltrating NK cells and the decrease in CD4^+^ T cells in tumours of H4R-KO mice was followed by an increment of CD19^+^ lymphocytes, that was observed at day 28 p.i. of 4T1 cells, even if they show very low expression of H4R.^55^ Infiltrating CD19^+^ B lymphocytes have been much less evaluated as prognostic or predictive factors in breast cancer. Their presence within breast lesions is about 20% of total TILs, and has been associated with more favourable prognosis due to the ability of these lymphocytes to differentiate into granzyme B-secreting cells.^6^

We hypothesise that the differences observed in the percentages of tumour-infiltrating B cells could be due to the interaction of NK cells and B cells through both indirect and direct pathways. NK cells lead to B cell activation and induce class switching of immunoglobulins produced through CD40–CD40 ligand interaction and through the production of various cytokines.^70^ Additionally, B cell secreted antibodies may lead to antibody-dependent cell-mediated cytotoxicity by NK cells.^70^ Interestingly and in line with this, negative associations between the percentages of NK cells and CD19^+^ lymphocytes and tumour weight were observed in TDLN exclusively in H4R-KO mice.

The spleen is involved in the systemic regulation of immunity. It has no afferent lymph vessels and collects its leukocytes directly from blood. Besides circulating immune cells that continuously migrate into and out of the resting spleen, different diseases can recruit additional cells to the organ.^27^ Spleen weight of H4R-KO mice was lower than the one of the WT mice, together with a reduced tumour size of the former animals. Although no differences were observed between groups regarding immune cell subsets distribution, the tumour weight negatively correlates with splenic CD4^+^, CD19^+^ and NK cell percentages only in H4R-KO mice. Therefore, it is possible that tumour growth and dissemination may be also regulated by H4R at a systemic level. Noteworthy, there is a decrease in the percentage of Tregs in spleens and non-draining LN of H4R-KO mice, which could favour the systemic immune responses, thus reducing tumour progression.

Present findings demonstrate that histamine through H4R plays important roles at a variety of stages during tumour development and in multiple cell types including cancer cells and immune cells. H4R not only may participate in the modulation of cancer cell proliferation but also in the response of tumour immunity further supporting the complexity of cancer. Current studies are focused on evaluating the immunomodulatory role of H4R in other cancer models.

As far as we know, this is the first report showing the participation of H4R in the anti-tumour immunity, highlighting the therapeutic potential of H4R ligands as adjuvants to cancer therapy, modulating immune response. Further studies are needed to determine the exact role of H4R in cancer biology, considering the triggered effects in H4R expressing cells in the tumour and in the immune tumour microenvironment, as a whole, in order to improve the benefit of cancer patients.

## Electronic supplementary material

Supplementary Table 1

Supplementary Table 2

Supplementary Table 3
