# Supplementary material for: Immunomodulatory role of histamine H4 receptor in breast cancer
Source: Br J Cancer. 2018 Jul 10;120(1):128–38. doi: 10.1038/s41416-018-0173-z (PMC6325108; doi:10.1038/s41416-018-0173-z)
Supplement: Supplementary file 2 — Supplementary Table 2 [file 41416_2018_173_MOESM2_ESM.docx]

Supplementary Table 2: Correlation coefficient (R) value of tumor weight (g) versus immune cells subset percentage in TDLN of WT and H4R-KO mice

|  | **WT** | | **KO** | |
| --- | --- | --- | --- | --- |
|  | **R** | ***P*** | **R** | ***P*** |
| **CD3^+^** | -0.2495 | P>0.05 | -0.4274 | P>0.05 |
| **CD4^+^** | -0.3208 | P>0.05 | -0.6274 | P>0.05 |
| **CD8^+^** | 0.2449 | P>0.05 | 0.7730 | 0.0714 |
| **CD3^-^CD49^+^** | 0.3577 | P>0.05 | **-0.8874** | **0.0183** |
| **CD19^+^** | -0.0587 | P>0.05 | **-0.8604** | **0.0279** |
