# Supplementary material for: Immunomodulatory role of histamine H4 receptor in breast cancer
Source: Br J Cancer. 2018 Jul 10;120(1):128–38. doi: 10.1038/s41416-018-0173-z (PMC6325108; doi:10.1038/s41416-018-0173-z)
Supplement: Supplementary file 3 — Supplementary Table 3 [file 41416_2018_173_MOESM3_ESM.docx]

Supplementary Table 3: Correlation coefficient (R) value of tumor weight (g) versus immune cells subset percentage in spleen of WT and H4R-KO mice

|  | **WT** | | **KO** | |
| --- | --- | --- | --- | --- |
|  | **R** | ***P*** | **R** | ***P*** |
| **CD3^+^** | -0.2903 | P>0.05 | **-0.8350** | **0.0194** |
| **CD4^+^** | -0.1741 | P>0.05 | **-0.9751** | **0.0002** |
| **CD8^+^** | -0.2458 | P>0.05 | 0.4053 | P>0.05 |
| **CD3^-^CD49^+^** | -0.0569 | P>0.05 | **-0.8616** | **0.0277** |
| **CD19^+^** | -0.3256 | P>0.05 | **-0.8503** | **0.0153** |
